# Draft Genome Sequence of Methanol-Utilizing *Methylophilus* sp. Strain OH31, Isolated from Pond Sediment in Hokkaido, Japan

**DOI:** 10.1128/genomeA.00274-14

**Published:** 2014-04-10

**Authors:** Takahiro Kugo, Wataru Kitagawa, Yoshinori Shimomura, Takuya Yamagishi, Michiko Tanaka, Teruo Sone, Kozo Asano, Yoichi Kamagata

**Affiliations:** a Graduate School of Agriculture, Hokkaido University, Sapporo, Japan; b Bioproduction Research Institute, National Institute of Advanced Industrial Science and Technology (AIST), Sapporo, Japan

## Abstract

*Methylophilus* sp. strain OH31 was isolated from the sediment of the Ohno pond at Hokkaido University. Strain OH31 utilizes methanol as its energy source. Here, we present the draft genome sequence of *Methylophilus* sp. strain OH31.

## GENOME ANNOUNCEMENT

Members of the genus *Methylophilus* comprise nonhalophilic, obligate, and restricted facultatively methylotrophic bacteria having the ribulose monophosphate pathway for formaldehyde assimilation. The ability to grow on methanol is common to the type strains of these species (1). At the time of writing, the genus comprised 7 recognized species (1–6). A few draft genome sequences within the genus have been deposited in the databases; however, none of them had been published in the literature at that time.

*Methylophilus* sp. strain OH31 is a methanol utilizer isolated from a methane-amended enrichment culture in which a methanotrophic bacterium coexisted as the primary contributor to methane consumption. We collected a sediment sample from Ohno pond (43°4.744′N, 141°20.793′E) at Hokkaido University (Sapporo, Japan). The sample was diluted with minimum salt medium (7–9) to 10× volume and incubated at 20°C in a closed vial containing air supplemented with 10% (vol/vol, headspace) methane. When the methane consumption was observed, 10% (vol/vol) of the culture liquid was transferred to fresh medium. In the same manner, the sample was subcultured repeatedly. After 1.5 months of subculture (11 consecutive cultures), the total DNAs of the microorganisms in the medium were extracted and subjected to community analysis based on 16S rRNA gene sequencing. The analysis revealed that the microbial community was composed mostly of *Methylomonas* spp. and *Methylophilus* spp. In order to isolate the *Methylophilus* organisms, a part of the liquid culture (11th generation) was then transferred to a gellan gum-solidified medium supplemented with methanol in vapor form. Strain OH31 was found to be the fastest grower on the medium.

In this study, the draft genome sequencing of strain OH31 was performed, and the genes involved in methanol oxidation activity were identified. The whole genome of strain OH31 was sequenced by paired-end sequencing on an Illumina HiSeq 2000 sequencing system provided by the Hokkaido System Science Co., Ltd. (Sapporo, Japan). This sequencing run yielded 19,852,162 high-quality filtered reads with 101-bp paired-end sequencing, providing approximately 690-fold genome coverage. Using the Velvet version 1.2.01 program with a hash length of 95 bp, these reads were assembled into 16 contigs, which had an average length of 183,293 bp. The prediction of protein coding sequences (CDS) and annotation were performed by the MiGAP pipeline version 2.17 (10), which utilizes MetaGeneAnnotator, RNAmmer, tRNAscan-SE, and BLAST (11–14). The draft genome sequence of strain OH31 comprises 2,932,698 bp, with a G+C content of 50.6%. The genome contains 2,757 putative CDS and 40 tRNAs.

The annotated genome sequences revealed 6 putative methanol dehydrogenase genes that show 55 to 98% estimated amino acid similarities with those of related methylotrophic bacteria. In addition to those genes, 2 putative formaldehyde dehydrogenase genes were identified.

This information helps not only in understanding the remarkable methanol-utilizing ability of OH31 but also in uncovering the role of methanol utilizers in general in methane-oxidizing environments.

### Nucleotide sequence accession numbers.

The draft genome sequence has been deposited at DDBJ/EMBL/GenBank under the accession no. BAUS00000000. The version described in this paper is the first version, BAUS01000000.
